# Reactive Synthesis for Porous (Mo_2/3_Y_1/3_)_2_AlC Ceramics through Mo, Y, Al and Graphite Powders

**DOI:** 10.3390/ma17133272

**Published:** 2024-07-02

**Authors:** Siwei Tan, Gan Xiao, Baogang Wang, Kui Yu, Jie Li, Wenkai Jiang, Heng Zhang, Xuejin Yang, Junsheng Yang

**Affiliations:** College of Mechanical Engineering, Wuhan Polytechnic University, Wuhan 430023, China; tansiweimin@163.com (S.T.); xiaogan202211@163.com (G.X.); wbg0217@163.com (B.W.); lj820680639@163.com (J.L.); jiangwenk@whpu.edu.cn (W.J.); zh20061100068@163.com (H.Z.); yangxuejin.2007@163.com (X.Y.); yangjunsheng2008@163.com (J.Y.)

**Keywords:** (Mo_2/3_Y_1/3_)_2_AlC, porous ceramics, activation reaction sintering, synthetic route

## Abstract

Through an activation reaction sintering method, porous (Mo_2/3_Y_1/3_)_2_AlC ceramics were prepared by Mo, Y, Al, and graphite powders as raw materials. The phase composition, microstructure, element distribution, and pore structure characteristics were comprehensively studied using X-ray diffraction (XRD), scanning electron microscopy (SEM), energy-dispersive spectroscopy (EDS), Archimedes method, and bubble point method. A detailed investigation was conducted on the influence of sintering temperature on the phase composition. Possible routes of phase transition and pore formation mechanisms during the sintering process were provided. The experimental results reveal that at 650–850 °C, transition metals react with aluminum, forming aluminum-containing intermetallics and a small amount of carbides. At 850–1250 °C, transition metals collaborate with graphite, producing transition metal carbides. Then, at 1250–1450 °C, these aluminum intermetallics interact with transition metal carbides and remaining unreacted Y, Al, and C, yielding the final product (Mo_2/3_Y_1/3_) _2_AlC. Simultaneously, the pore structure alters correspondingly with the solid-phase reaction at different reaction temperatures.

## 1. Introduction

In 2000, Barsoum et al. [1] conducted a systematic review of ternary layered transition metal carbides or nitrides for the first time, and proposed the concept of “M_n+1_AX_n_”, as a general term for this newly emerging ceramic material. Among them, n is usually equal to 1–3, M is an early transition metal, A is an A-group element, and X is C or N. According to the difference in n, MAX can generally be divided into 211, 312, and 413 types [2,3]. Due to the ionic or covalent bond between M-X and the metallic bond between MX and A layers, MAX phase ceramics combine the unique properties of both ceramics and metals, such as machinability, ductility, electrical conductivity, thermal conductivity, good oxidation resistance, etc. [4,5,6].

The self-propagating high-temperature synthesis (SHS) technique is a simple and effective technology in the synthesis of MAX phase substance. Hashimoto et al. [7] investigated the mechanism of Ti_2_AlC formation from 2Ti:1Al:1C powder during a self-propagating high-temperature synthesis (SHS) process. Firstly, Ti and C undergo an exothermic reaction to generate TiC after ignition, causing Al to melt and form an Al-Ti liquid phase. Subsequently, TiC dissolves into the Al-Ti liquid phase, forming Ti_2_AlC. Thomas et al. [8] also confirmed the formation process of Ti_2_AlC and investigated the effect of carbon source particle size on the phase composition of 2Ti:1Al:1C powder samples using the SHS technique. When using carbon fiber as a carbon source, due to the shape and size of the carbon fiber, the contact area between Al and Ti increases, resulting in a high thermal conductivity of the cold-pressed reactants. Ti_3_Al alloy and TiC rapidly form and then rapidly cool, so the high-temperature insulation time is insufficient to allow Ti-Al and TiC to form Ti_2_AlC. When graphite is used as a carbon source, the contact area between Al and Ti is decreased due to being surrounded by carbon, resulting in the low thermal conductivity of the cold-pressed reactants, which greatly slows down the speed of the combustion wave and ensures the complete reaction between Ti-Al and TiC, finally generating Ti_2_AlC. However, SHS technology has certain limitations. Firstly, the reaction must be highly exothermic. Secondly, reactants can form liquid or gaseous states during the reaction process for the diffusion and transport of substances [9]. As for the synthesis of the MAX phase, SHS is a complex multi-step process that is difficult to control, and impurity phases are inevitably formed in the product [10,11].

In addition to SHS, hot pressing (HP) is also a method for synthesizing MAX phase. Zhu et al. [12] synthesized dense Ti_2_AlN ceramics using the hot pressing method under a nitrogen atmosphere and explored the formation mechanism. The experiment shows that Ti_2_N, TiAl, and Ti_3_Al phases are mainly present at 500 °C. As the temperature increases, Ti_2_N and TiAl decrease, and Ti_2_AlN begins to appear. At 1200 °C, except for a small amount of TiN, it is mainly composed of Ti_2_AlN. Therefore, in the hot-pressed Ti-Al-N system, TiN and Ti-Al alloys (Ti_3_Al, TiAl) were observed to form first and then react to synthesize Ti_2_AlN. Although HP is a mature method, it has certain limitations due to its complex process and equipment, high energy consumption, low production efficiency, and high production cost [13]. Usually, it is widely used in the synthesis of dense bodies.

Similar to HP, spark plasma sintering (SPS) typically applies pressure to powder compacts to enhance density [14]. In the study of Oh et al. [15], when preparing Cr_2_AlC from 2Cr:1.2Al:1C powder through SPS, Cr_7_C_3_ and Cr-Al alloys (Al_8_Cr_5_, AlCr_2_) were firstly formed at 700–800 °C and then reacted to generate Cr_2_AlC.

Furthermore, the activation reaction sintering methodology is a potent technique for preparing MAX phase materials. Concurrently, by setting up different insulation stages, the synthesis of intermediate phases can be realized, thereby diminishing the final sintering temperature. (Mo_2/3_Y_1/3_)_2_AlC and its MXene substances have been validated to possess substantial application prospects in the fields of electrochemical energy storage and electrocatalysis [16]. Nonetheless, the reaction process and synthesis mechanism of Y-containing MAX phase (Mo_2/3_Y_1/3_)_2_AlC, as well as the pore structure evolution law of the activation reaction sintering process, are still unclear. Therefore, the study of its reaction path has important reference significance for the subsequent preparation of similar Y-containing MAX substances.

In this paper, porous (Mo_2/3_Y_1/3_)_2_AlC ceramics were prepared by mixing and pressing four element powders of Mo, Y, Al and C through the utilization of vacuum activation reaction sintering technology. The effects of varying sintering temperatures on the phase structure evolution and reaction path were primarily discussed, and the pore formation mechanism was also analyzed.

## 2. Experimental Section

Commercially available Mo (99.6% pure, 3.5 μm), Y (99.6% pure, 3.5 μm), Al (99.6% pure, 3.5 μm) and graphite (99.6% pure, 3.5 μm) powders were used as the raw material and added to the planetary mill in an atomic ratio of Mo:Y:Al:C = 1.33:0.66:1.10:1. Then, anhydrous ethanol was added and stirred slowly with a glass rod to achieve an initial uniform dispersion of the raw material powders. Next, zirconia balls were added in a 5:1 (ball to materials) ratio in a planetary ball mill at 200 rpm for 48 h to ensure sufficient and uniform mixing of raw material powder. After the mixing was completed, the mixed slurry was dried in a vacuum drying oven at 50 °C, and finally, the required raw materials were obtained through a 200 mesh sieve. The mixed powder was pressed into a Φ25 mm green compact under a pressure of 160 MPa. To investigate the formation mechanism of porous (Mo_2/3_Y_1/3_)_2_AlC ceramics, the raw material was placed in a vacuum sintering furnace, and the sintering temperature was continuously increased from 650 °C to 1450 °C at intervals of 200 °C. The holding time for each stage was 2 h, and the heating rate was limited to 5 °C/min with a temperature deviation of ±5 °C.

The composition of the material was detected using X-ray diffraction (XRD, Dmax 2500VB, Rigaku Corp, Tokyo, Japan) with a scan rate of 5 °/min and a step size of 0.02°. The pore morphology was characterized using scanning electron microscopy (SEM, Zeiss SIGMA Field Emission SEM, Oberkochen, Germany). Energy-dispersive spectrometer (EDS) mapping was used to investigate whether the composition of the final sample was uniform. Porosity and pore size were determined using the Archimedes method and bubble point method to explore the evolution law of the pore structure [17,18].

## 3. Results and Discussion

### 3.1. Macroscopic Comparison of Samples

**Figure 1:**
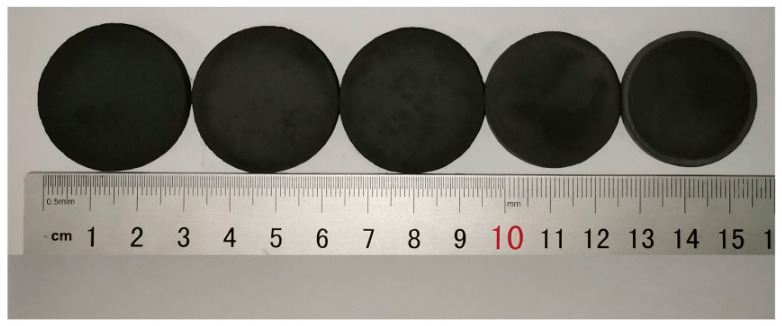
The macro morphology of the samples of different sintering temperatures: 650 °C to 1450 °C from left to right, with intervals of 200 °C.

Figure 1 illustrates the macroscopic morphology of porous (Mo_2/3_Y_1/3_)_2_AlC ceramics obtained at different sintering temperatures. The sintering interval was 200 °C, and the sintering temperatures of the samples from left to right were 650 °C, 850 °C, 1050 °C, 1250 °C, and 1450 °C, respectively. As shown in Figure 1, the overall volume change of the samples displays different trends at different temperatures. Overall, as the temperature increased, the sample exhibited a significant trend of radial shrinkage. Specifically, the shrinkage was most significant from 1050 °C to 1250 °C, while the changes were not significant at 650 °C to 1050 °C and 1250 °C to 1450 °C. Additionally, the sample surfaces remained relatively flat and smooth, with some spots caused by carbon from the furnace adhering to the surface. Other than that, there were no protrusions or fractures.

### 3.2. Effects of Sintering Temperature on the Synthesis of Porous (Mo_2/3_Y_1/3_)_2_AlC Ceramics

**Figure 2:**
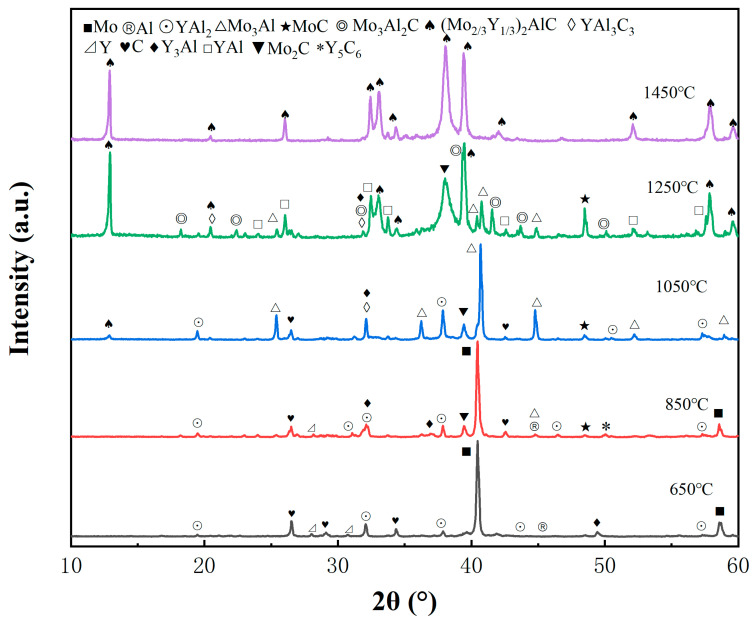
XRD diffraction patterns of (Mo_2/3_Y_1/3_)_2_AlC sintered at different temperatures.

Figure 2 presents the XRD pattern of the mixed powder with an atomic ratio of Mo:Y:Al:C = 1.33:0.66:1.10:1 during sintering.

Except for the Mo, Y, Al, and C elements that did not fully react, Y gradually reacted with solid Al, generating a small number of YAl_2_ at 650 °C. Upon raising the sintering temperature i to 850 °C, there was a significant increase inYAl_2_, accompanied by the formation of intermetallic compounds such as Y_3_Al and Mo_3_Al, as well as the initial appearance of Mo_2_C and Y_5_C_6_ carbides. In the study by Yang et al. [19], it was found that Ti-C compounds have a lower enthalpy of formation compared to Ti-Al compounds, suggesting Ti-C compounds should theoretically form more readily. However, experimental results show that Ti-Al compounds are more easily observed. Similarly, in this experiment, when the temperature was raised to 850 °C, intermetallic compounds of Y-Al and Mo-Al were observed, with only a small amount of carbides present. As the sintering temperature further increased to 1050 °C, characteristic peaks of MAX phase appeared at low angles, indicating that a small amount of (Mo_2/3_Y_1/3_)_2_AlC had already been generated. As carbides such as Mo_2_C and MoC gradually increased, YAl_2_ also steadily increased. Moreover, the content of Mo_3_Al increased sharply, becoming the dominant phase at this temperature. Additionally, a small amount of Y-C compound began to slowly combine with Y-Al to form a new phase, YAl_3_C_3_.

Continuing to increase the sintering temperature to 1250 °C, the characteristic peak of the MAX phase at low angles significantly increases. Additionally, an increasing amount of (Mo_2/3_Y_1/3_)_2_AlC can be observed in the figure. As carbides increase, the peaks of Mo_3_Al and YAl_3_C_3_ decrease, and new substances identified as Mo_3_Al_2_C and YAl emerged. Therefore, the increased presence of Mo-C carbides not only promotes the generation of the transitional phase Mo_3_Al_2_C, but also initiates the transition of some YAl_3_C_3_ towards the final MAX phase. It can be preliminarily concluded that the sintering temperature of 1250 °C represents a critical period before the formation of the MAX phase (Mo_2/3_Y_1/3_)_2_AlC. At this temperature, Mo-Al, Mo-C, Y-Al, and Y-C compounds interact to form key early-transition phases Mo_3_Al_2_C and YAl_3_C_3_, essential for synthesizing (Mo_2/3_Y_1/3_)_2_AlC. It is worth noting that Aliakbari et al. [20] found that Y_2_AlC showed the lowest stability in the Y-series 211 MAX phase, and no corresponding 211-type MAX phase was observed in this experiment.

At the final sintering temperature of 1450 °C, pure phase (Mo_2/3_Y_1/3_)_2_AlC was successfully synthesized. Based on XRD analysis, the possible reaction pathways leading to (Mo_2/3_Y_1/3_)_2_AlC are as follows:

$$\begin{array}{c} \left.\begin{array}{c} \begin{array}{c} 2\mathrm{Mo}+C={Mo}_{2}C\\ \mathrm{Mo}+C=\mathrm{Mo}C\\ 3\mathrm{Mo}+\mathrm{Al}={Mo}_{3}\mathrm{Al}\\ Y+2\mathrm{Al}=Y{Al}_{2}\\ 3Y+\mathrm{Al}=Y_{3}\mathrm{Al} \end{array}\\ 5Y+C6=Y_{5}C_{6} \end{array}\right\}\;\;\;\;\;\;\;\;\;\;650-850\;{}^{\circ}C \end{array}\;$$

$$\begin{array}{c} \left.\begin{array}{cc} 3\mathrm{MoC}+3{Mo}_{3}\mathrm{Al}+5\mathrm{Al}+4C= & 4{Mo}_{3}{Al}_{2}C\\ Y_{3}\mathrm{Al}+25\mathrm{Al}+18C+Y_{5}C_{6}= & 8Y{Al}_{3}C_{3}\\ Y{Al}_{2}+Y= & 2\mathrm{YAl}\\ Y_{3}\mathrm{Al}+3\mathrm{Al}= & 3\mathrm{YAl} \end{array}\right\}\;\;\;\;\;\;\;850-1250\;{}^{\circ}C \end{array}$$

2Mo3Al2C+2MoC+2Y+YAl3C3+Y3Al=2YAl+C+6Mo23Y132AlC6Mo2C+6YAl+3C+3Al=9Mo23Y132AlC       1250−1450 °C

**Figure 3:**
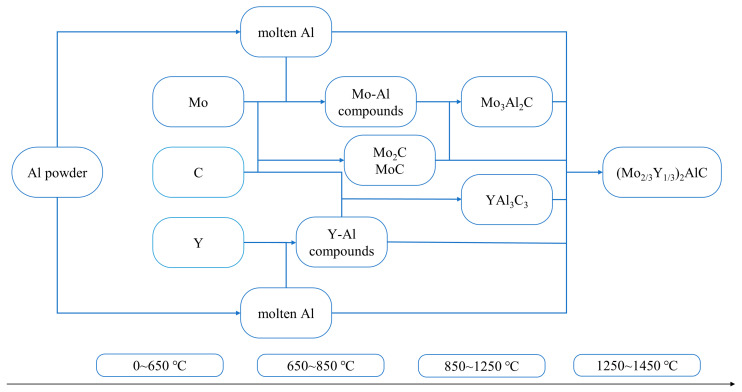
Schematic diagram of the overall reaction process.

The complete synthesis process diagram and phase composition are summarized in Figure 3 and Table 1.

### 3.3. Effects of Temperature on Volume Evolution and Pore Structure Parameters

Parameters related to pore structure are crucial for studying porous materials, both in terms of functionality and phase transition. Therefore, it is necessary to explore the changes in the pore structure of samples at different sintering temperatures.

**Figure 4:**
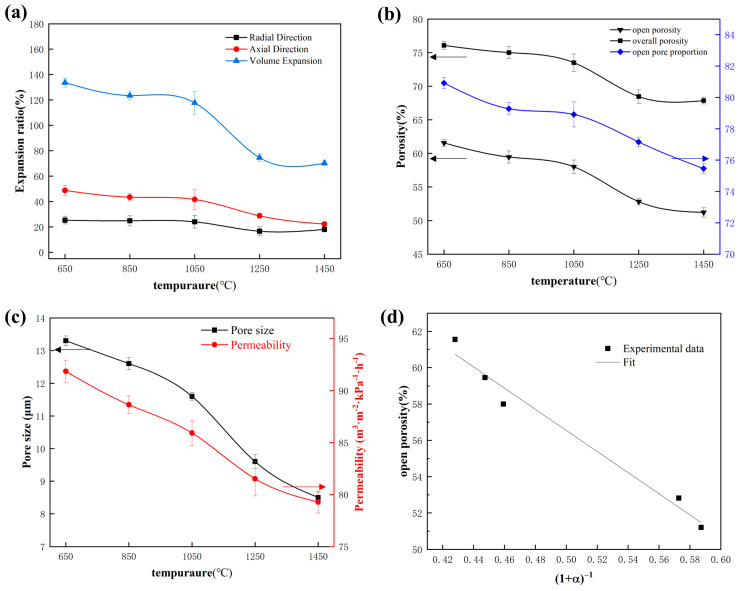
The effect of different sintering temperatures on volume expansion and pore structure parameters. (**a**) Volume expansion ratio; (**b**) porosity; (**c**) pore size and permeability; and (**d**) relationship between the volume expansion ratio and the open porosity.

Figure 4a illustrates the radial, axial, and volume variation trends of samples at different sintering temperatures. It can be observed that the overall volume expansion rate exhibits a trend of shrinkage, with a volume expansion rate of 133.63% at 650 °C. As the temperature increases, the volume expansion rate gradually decreases to 117.74% at 1050 °C. During this stage, the volume change of the sample is not significant. Based on the XRD pattern in Figure 2, the generation of intermetallic compounds and carbides predominantly occurs between 650 and 1050 °C. The excess unreacted molten Al reacts with Mo and Y to generate YAl_2_ and Mo_3_Al, with a slight shrinkage of the volume attributed to the surface tension of the liquid phase. Additionally, carbides such as MoC and Mo_2_C increase with temperature. Since carbide formation occurs through solid-phase reactions, these carbides compress the pores, resulting in a decrease in porosity and pore size. Based on the above theory, it can be inferred that the partial diffusion of Al and the generation of carbides jointly redistribute the pores, resulting in a gradual shrinkage trend in the final sample volume. When the temperature increased from 1050 to 1250 °C, there was a significant decrease in the volume expansion rate, dropping from 117.74% to 74.59%. Combined with Figure 2, a substantial number of MAX phases were generated during this period, along with intermediate transition phases YAl, Mo_3_Al_2_C, and YAl_3_C_3_. This indicates that the temperature range of 1050 °C to 1250 °C is a critical temperature node for MAX phase generation. On the one hand, carbides continued to increase, while on the other hand, a large amount of MAX phase was produced, resulting in severe volume shrinkage. When the final temperature rose to 1450 °C, the volume expansion rate decreased further to 70.29%, and the sample continued to shrink slowly. However, these changes are not distinguishable in the macroscopic morphology. Based on the XRD results, the generation of MAX mainly occurred between 1250 and 1450 °C. During this temperature range, the reaction process was relatively moderate, resulting in insignificant volume changes in the sample.

Figure 4b presents the trend of porosity variation in samples at different sintering temperatures. It is obvious that the trend of porosity change is consistent with the trend of volume expansion rate, with open porosity significantly decreasing from 58% at 1050 °C to 52.82% at 1250 °C. It is worth noting that the porosity of the unsintered compact was 17.4%. Despite the continuous shrinkage of the sample volume and gradual decreases in porosity during the sintering process, a large number of pores were still generated, resulting in a higher porosity than the green compact. The process of pore formation will be explained in detail in the section on pore-formation mechanism.

Figure 4c exhibits the trend of changes in pore size and permeability. The pore size also shows a steady decrease followed by a rapid decrease, and the permeability follows a similar trend. These observations indicate significant changes in the internal structure of the sample between 1050 °C and 1250 °C, resulting in drastic changes in the pore structure parameters of porous (Mo_2/3_Y_1/3_)_2_AlC ceramics during this stage. This is externally manifested as the sharp shrinkage of the sample at 1250 °C, as shown in Figure 1. The transformation process will be elaborated in detail in the next section.

Figure 4d reveals a linear fitting graph between the porosity and expansion rate. It can be seen from the graph that there is still a strong linear relationship between the volume expansion rate and porosity. The fitting formula is as follows [17]:

$$\theta_{p}=\frac{\rho_{0}\left(1-\theta_{0}\right)}{\rho }\frac{1}{1+\alpha }+\left(1-\theta_{c}\right)$$

where $\rho_{0}$ and ρ are the theoretical densities of the compact before and after the sintering, $\theta_{0}$ is the initial overall porosity in green compacts, $\theta_{p}$ is the open porosity after the sintering, α is the volume expansion ratio, and $\theta_{c}$ is the closed porosity in the sintered compact.

### 3.4. Micromorphology of Porous (Mo_2/3_Y_1/3_)_2_AlC Ceramics

**Figure 5:**
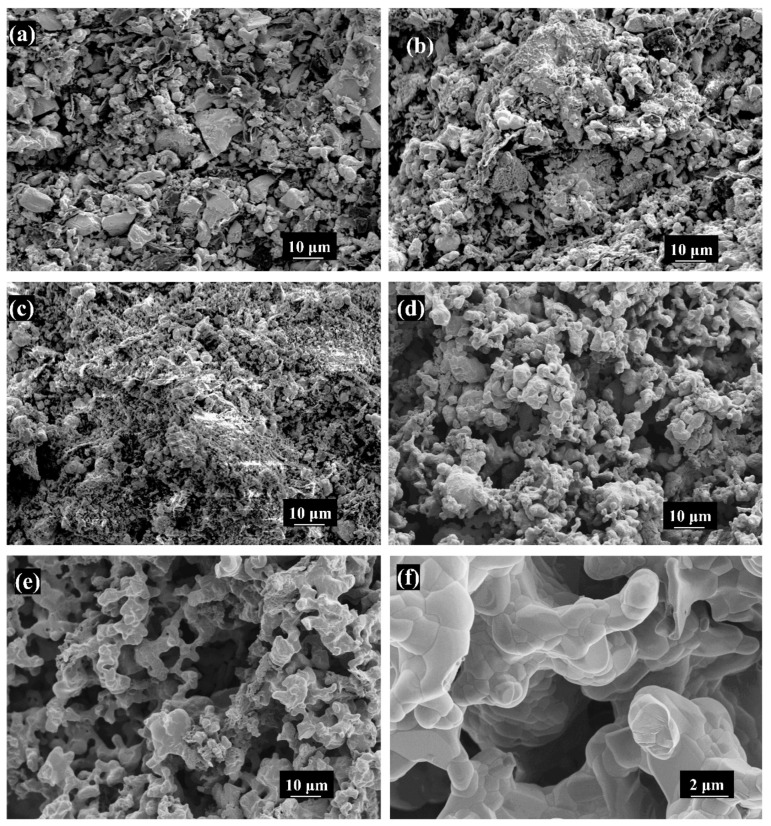
Scanning electron microscopy images at different sintering temperatures: (**a**) 650 °C, (**b**) 850 °C, (**c**)1050 °C, (**d**) 1250 °C, (**e**) 1450 °C and (**f**) higher magnification images under 1450 °C.

Figure 5 exhibits the scanning electron microscopy images of porous (Mo_2/3_Y_1/3_)_2_AlC ceramics at temperatures ranging from 650 °C to 1450 °C.

Based on the XRD patterns in Figure 2 and Table 1, it can be seen that in Figure 5a, although the Al element forms a small amount of YAl_2_ and Y_3_Al with a small amount of Y, Mo remains the dominant phase, and irregular granular particles still dominate the morphology. Similarly, when the temperature rises to 850 °C, Mo still occupies the main position, but due to the increase in YAl_2_ and the appearance of carbides such as Mo_2_C, MoC, and Y_5_C_6_, fused small and smooth particles can be clearly seen on the surface of the sample. When the temperature is further raised to 1050 °C, the peak of Mo disappears, and except for graphite(C), all other elemental powders are no longer detected. Instead, as YAl_2_ slowly increases, Mo_3_Al becomes the dominant phase. In addition, the newly appeared YAl_3_C_3_, combined with slightly increased Mo-C carbides, presents a flocculent morphology, adhering to the surface of the sample. Combined with the macroscopic morphology analysis in Figure 1, it can be seen that before 1050 °C, the overall volume expansion rate of the sample showed an almost imperceptible shrinkage trend, indicating that the reaction was relatively moderate before 1050 °C and had little impact on the overall morphology of the sample.

**Figure 6:**
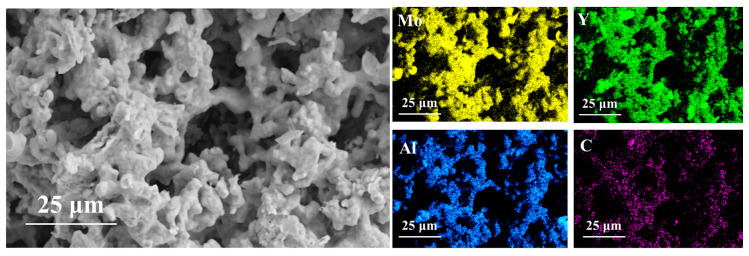
EDS elemental mapping images of porous (Mo_2/3_Y_1/3_)_2_AlC.

As mentioned in the previous analysis, 1250 °C is the critical temperature for the formation of porous (Mo_2/3_Y_1/3_)_2_AlC ceramics. Figure 5d exhibits that particle connections become more pronounced, and the surface gradually becomes smooth. In Figure 1, the sample volume shows significant shrinkage at the macro level, while at the micro level, Kirkendall pores become clear and visible. At a larger magnification, as shown in Figure 5f, it is evident that the particles are tightly bound and exhibit a distinct layered structure. Additionally, Figure 6 presents an EDS mapping map at the final sintering temperature of 1450 °C. The mapping shows that the distribution of each element is uniform, with no segregation, indicating that the raw materials were well mixed and the fusion between various elements was homogeneous after sintering.

### 3.5. Pore-Forming Mechanism

The changes in the volume of porous materials and the evolution of pore structure are closely related to the mechanism of pore formation. Studying this mechanism can provide a theoretical basis for the subsequent regulation of the pore structure of porous materials. Therefore, this section focuses on elucidating the pore formation mechanism of porous (Mo_2/3_Y_1/3_)_2_AlC ceramics.

Firstly, during the preparation process, when the mixture of Mo, Y, Al, and graphite powders was pressed into a green compact under a pressure of 160 MPa, there would have been certain gaps between the powder particles, which is one of the reasons for the formation of pores. Secondly, the XRD pattern presented in Figure 2 indicates that at 650 °C, the elemental powders had not yet fully reacted, while Al gradually began to melt into a liquid phase and flowed around. In previous reports [21], regardless of whether the (Cr, Y)AlC or (W, Y)AlC system was used, after the formation of the (M′M″AX) phase, impurity phases always appear in the form of $M_{x}^{\prime }C_{y}$ and $M_{x}^{\prime \prime }{\mathrm{Al}}_{y}$, and the presence of $Y_{x}C_{y}$ is almost not observed. It is speculated that Y preferentially reacts with Al. Combined with the research of Watson et al. [22], YAl_2_ is identified as a stable phase, whereas Y_3_Al is considered a metastable phase. According to Chen et al. [23], in the Ti-Al system, due to the higher diffusion rate of Al compared to Ti, the net movement and consumption of Al elements must be balanced by opposite vacancies, resulting in excessive vacancies. To reduce the Gibbs free energy of the system, Kirkendall pores form by the condensation of supersaturated vacancies, replacing the original positions of the metal Al. This process is manifested by the rapid diffusion and consumption of Al elements, along with the aggregation and collapse of vacancies.

When the temperature rose to 850 °C, the content of YAl_2_ increased significantly, and a small amount of intermetallic compounds Y_3_Al and Mo_3_Al were generated. This indicates that molten Al continued to flow towards Mo, reacting with synthesized MoAl_3_, causing a slight volume contraction. As the temperature continued to rise to 1050 °C, elemental Mo disappeared and was replaced by Mo_3_Al as the main phase. Simultaneously, the content of YAl_2_ and Y_3_Al also steadily increased, and the carbides also gradually increased. Therefore, it can be inferred that molten Al rapidly moved towards Y and Mo, gradually enveloping Y and Mo and forming corresponding intermetallic compounds near Y and Mo, leaving vacancies and generating pores. However, the increasing content of Mo_2_C and MoC carbides indicates that the solubility of carbides gradually decreased, squeezing the original pores. In addition, the fusion between elements during the reaction led to a slight volume shrinkage. Based on the macroscopic morphology in Figure 1 and the pore structure changes in Figure 4, both the volume expansion rate and porosity show a slight downward trend overall. Between 650 °C and 1050 °C, the formation and transformation of phases were generally predominantly driven by the flow of liquid-phase Al. Molten Al reacted with Mo and Y to generate corresponding intermetallic compounds, and the surface tension of liquid Al promoted the formation of pores during this stage. This also explains why, despite the decreasing trend in porosity shown in Figure 4, the overall porosity remained higher than that of the green compact.

When the temperature rises to 1250 °C, the characteristic peaks of the MAX phase at low angles are significantly enhanced, and peaks of (Mo_2/3_Y_1/3_)_2_AlC at 32.48°, 33.1°, 39.46°, and 57.84° begin to appear, indicating that a substantial amount of the MAX phase was formed at this time. In addition, YAl_2_ disappeared while YAl increased. Based on previous analysis, metastable Y_3_Al and unstable yttrium–carbon carbides fuse to form the transition phase YAl_3_C_3_, and MoC combines with Mo_3_Al to form Mo_3_AlC_2_. These transition phases reacted with YAl to generate a significant amount of the MAX phase, resulting in an increase in carbides. The generation of the MAX phase led to strong volume shrinkage at the macroscopic level and a significant decrease in porosity and pore size at the microscopic level. Finally, at the final sintering temperature of 1450 °C, all reactions were essentially completed, and the corresponding volume expansion rate and pore-change trend stabilized at a relatively consistent level.

## 4. Conclusions

Though samples were prepared at different sintering temperatures ranging from 650 °C to 1450 °C at intervals of 200 °C, the phase transition of porous (Mo_2/3_Y_1/3_)_2_AlC ceramics during the synthesis process was investigated, and possible reaction pathways were provided. The results of EDS mapping indicate that each element was evenly distributed; a detailed explanation of the pore formation mechanism is provided. In addition to the pores left during cold pressing, the formation of pores at 650 °C was mainly due to the difference in diffusion rates between solid-phase Al and solid-phase Mo and Y, resulting in Kirkendall pores. From 650 °C to 1050 °C, excess unreacted molten Al flowed to Mo and Al, forming intermetallic compounds. The sample shrank slightly due to the surface tension of the liquid Al. From 1050 °C to 1450 °C, phase transformation was dominant, and the generation of carbides and MAX phases led to the severe shrinkage of the sample, thereby achieving the redistribution of pores. This research work could provide a reference for the preparation of other porous Al-containing MAX phases materials.

## Figures and Tables

**Table 1:** Summary of phase constitution of the samples heated in the different sintering temperatures.

Temperature	Phase	Main Phase
650 °C	Mo, Y, Al, C, YAl_2_	element powder
850 °C	Mo, Y, Al, C, YAl_2_, Y_3_Al, Mo_3_Al, Y_5_C_6_, Mo_2_C, MoC	element powder
1050 °C	Mo_3_Al, YAl_2_, Y_3_Al, Mo_2_C, MoC, YAl_3_C_3_, (Mo_2/3_Y_1/3_)_2_AlC, C	Mo_3_Al, YAl_2_
1250 °C	(Mo_2/3_Y_1/3_)_2_AlC, YAl, Mo_2_C, MoC, YAl_3_C_3_, Mo_3_Al_2_C, Y_3_Al, Mo_3_Al	YAl, Mo_2_C, (Mo_2/3_Y_1/3_)_2_AlC
1450 °C	(Mo_2/3_Y_1/3_)_2_AlC, Mo_3_Al_2_C, YAl_3_C_3_, Y_3_Al, YAl, Mo_2_C, MoC, Mo_3_Al	(Mo_2/3_Y_1/3_)_2_AlC

## Data Availability

Data are contained within the article.
